# News and Notes

**Published:** 1903-03

**Authors:** 


### *NEWS AND NOTES.*

(We are indebted to the *Medical Age* for many of these notes.)

---

THE prefect of the Seine having placarded Paris with posters describing the terrible effects of alcohol and absinthe drinking, the café proprietors each filed a damage suit against him.

---

THE Manila Medical Society has been organized in Manila, P. I., along the lines proposed for local organization by the American Medical Association, and has made formal application for recognition as an affiliated body.

---

THE recently ascertained fact that pearls are formed as the result of irritation of the oyster from a worm which is its parasite has led to the suggestion that beds of pearl oysters be infected with the worms and cultivated during the time necessary for pearls to develop. A mollusk's hospital is an altogether new idea.

---

THE physical director at Yale College has measured all students entering in the last nine years. The non-smokers average fifteen months younger than the smokers, are taller, and during the four years in school gain 24 per cent. more in height and 26.7 per cent. more in chest growth than do habitual users of tobacco.

---

OF the 165 kinds of snakes found in the United States, but twenty are venomous. They are the copperhead and water-moccasin, which are closely related; the coral snakes of the southwest, and the two species of sistrurus and the fifteen species of rattlesnakes. The most dangerous of them, the water-moccasin, is not seen north of Tennessee.

CHIEF Medical Inspector of Chicago, Dr. Spaulding, says: "If parents could see, as I am compelled to see, sorrowful mothers walking the floor with festering children in their arms, crying and reproaching themselves for neglecting to have their little ones vaccinated, there would be no need of urging the vaccination of babies. A vaccinated child never was known to have smallpox."

---

THE following grants of money to assist original research by members of Johns Hopkins University have been made by the Carnegie Institute: For measurement of osmotic pressure, $1,500; for a research assistant to Professor Wood, $1,000; for physical chemistry, $1,000; apparatus for physiological chemistry research, $1,000; for research in the theory of a magnetic field, amount not fixed.

---

"FOR the collection of sociological and pathological data, especially such as may be found in institutions for the criminal, pauper and defective, and generally in hospitals and schools," is the title of a bill recently introduced into Congress. The United States provides for the punishment of crime, but makes no effort to prevent it. The annual tribute society pays to crime, statistics show to be $600,000,000.

---

THE laments of eminent personages over the decreasing birth rate in the United States have prompted Chicago's health department to print in its bulletin the best possible evidence that the birth rate in that city is satisfactory. It says: "The increase in the under seven-year population was 57.3 per cent., or 5.3 in excess of the increase of the total population at all ages. This excess is obviously due to the births during the period."

---

THE next meeting of the American Medical Association will be held in New Orleans, May 5, 6, 7 and 8, 1903. The general officers are: President, Frank Billings, Illinois; First Vice-President, J. A. Witherspoon, Tennessee; Second Vice-President, G. F. Comstock, New York; Third Vice-President, C. R. Holmes, Ohio; Fourth Vice-President, James H. Dunn, Minnesota; Secretary-Editor, George H. Simmons, Illinois; Treasurer, Henry P. Newman, Illinois; Chairman Committee of Arrangements, Isadore Dyer, 124 Baronne Street, New Orleans, La.

---

THE Suez Canal Company asked to have Dr. Ross sent out to suggest measures to combat malaria at Suez. The local physicians had been studying the cause why mosquitoes were so prevalent in that arid country, although there seems to be no stagnant water for them to hatch in. They found that although the surface is dry, yet there is moisture close to the surface in many places, forming concealed marshes, revealed only by occasional tufts of grass. They found the Anopheles at these points, and Ross has already instituted measures for their destruction on the lines that have been so successful in Western Africa.—*Journal of the American Medical Association.*

---

IT is announced by the *New York Medical Journal* that Henry Phipps, of New York, once a partner of Andrew Carnegie, will establish in Philadelphia an institute for the study, treatment, and prevention of tuberculosis, and will endow it with $1,000,000 or more. It will be named the Henry Phipps Institute, and will be located in the center of the city among the poor, if a suitable site can be obtained, where it will be easily accessible, and where the best scientific talent can be obtained for the various positions in the institute. Dr. Lawrence Flick, of Philadelphia, will be the director. The institute will be an entirely independent charity. The buildings will cost probably between $200,000 and $300,000, and the endowment will be $1,000,000 or more to yield an income of about $40,000 a year for the institute's maintenance.

---

THE only Chinese hospital with Chinese doctors, Chinese medicine, Chinese nurses, and Chinese patients, is situated on the border of Chinatown, San Francisco. It is, according to the *Philadelphia Medical Journal*, a large new two-story building, and has but recently been erected. Up to this time a Chinese, unless afflicted with leprosy, in which case he was sent to the pest-house, was not admitted to the public hospitals. Rich Chinese merchants subscribed $30,000, the income of which supports the present Chinese hospital. There is a daily clinic held in the hospital, with a resident white physician and surgeon; there is also a Chinese department with a Chinese doctor. Patients entering are told to choose European treatment. As many as 400 cases are treated in a month, with only fifteen deaths. The hospital contains forty beds.

---

THE New Orleans meeting of the American Medical Association will take place May 5 to 8. The Southern Railway has announced a reduced rate of one fare for the round trip from Washington, or from any point on their system, to New Orleans and return. Tickets will be on sale May 1 to 4, and will be good for continuous passage in each direction, with a final limit of ten days from the date of sale. Tickets can be extended for a longer period, however, provided they are deposited in person, by the original purchaser, with the special agent at New Orleans not later than May 12, 1903, and fee of 50 cents is paid at the time of deposit, when the final limit will be extended to a date not later than May 30. Further information may be obtained from the chairman of the committee on transportation, Dr. H. L. E. Johnson, Jefferson Place, Washington, D. C.

